# Retraction: MiR-320d suppresses the progression of breast cancer *via* lncRNA HNF1A-AS1 regulation and SOX4 inhibition

**DOI:** 10.1039/d2ra90088b

**Published:** 2022-09-20

**Authors:** Shuai Shi, Xiaoling Hu, Jianpo Xu, Hong Liu, Libo Zou

**Affiliations:** Reproductive Medicine Center of Jinhua People's Hospital, Biomedical Research Center of Zhejiang Normal University Jinhua 321000 China Shuai5189188@qq.com +(86) 18258950539; Department of Reproductive Endocrinology, Zhejiang University School of Medicine Affiliated Obstetrics and Gynecology Hospital Hangzhou 310006 China; Life Sciences Institute of Zhejiang University Hangzhou 310058 China

## Abstract

Retraction of ‘MiR-320d suppresses the progression of breast cancer *via* lncRNA HNF1A-AS1 regulation and SOX4 inhibition’ by Shuai Shi *et al.*, *RSC Adv.*, 2018, **8**, 19196–19207, https://doi.org/10.1039/C8RA01200H.

The Royal Society of Chemistry hereby wholly retracts this *RSC Advances* article due to concerns with the reliability of the data.

Many of the published western blot panels contain duplicating features between the backgrounds of different panels, indicating that they have been manipulated. For example, there are duplicating features between the backgrounds of the panels in Fig. 6E (c-myc/MCF-7) and Fig. 7B (SOX4/MDA-MB-231). There are also duplicating features between the backgrounds of the SOX4/MCF-7 and SOX4/MDA-MB-231 panels in Fig. 7B.

Three of the bands shown in Fig. 7D (β-actin MCF-7 blots 1 and 4, and β-actin MDA-MB-231 blot 3) also contain duplicating features, indicating that they have been artificially generated. There are also repeating fragments in the backgrounds of the two panels.

The authors were asked to provide the raw data for this article, but did not respond. Given the significance of the concerns about the validity of the data, and the lack of raw data, the findings presented in this article are not reliable.

The authors were informed but have not responded to any correspondence regarding the retraction.

Signed: Laura Fisher, Executive Editor, *RSC Advances*

Date: 18th August 2022

## Supplementary Material

